# Effects of a Herbal Medicine, Yukgunja-Tang, on Functional Dyspepsia Patients Classified by 3-Dimensional Facial Measurement: A Study Protocol for Placebo-Controlled, Double-Blind, Randomized Trial

**DOI:** 10.1155/2017/2894507

**Published:** 2017-03-14

**Authors:** Juyeon Kim, Jae-Woo Park, Seok-Jae Ko, Soo-Hyung Jeon, Jong-Won Kim, Inkwon Yeo, Jinsung Kim

**Affiliations:** ^1^Department of Gastroenterology, College of Korean Medicine, Kyung Hee University, Kyungheedae-ro 26, Dongdaemun-gu, Seoul 02447, Republic of Korea; ^2^Department of Sasang Constitutional Medicine, College of Korean Medicine, Dong-Eui University, 62 Yangjeong-ro, Busanjin-gu, Busan 47227, Republic of Korea; ^3^Department of Statistics, Sookmyung Women's University, Cheongpa-ro 47-gil 100, Youngsan-gu, Seoul 140-742, Republic of Korea

## Abstract

*Introduction*. Functional dyspepsia (FD), a common upper gastrointestinal disease, is difficult to manage because of the limitations of current conventional treatments. Yukgunja-tang (YGJT) is widely used to treat FD in clinical practice in Korea, Japan, and China. However, YGJT significantly improves few symptoms of FD. In Korean medicine, FD is a well-known functional gastric disease that shows difference in the effect of herbal medicine depending on constitution or type of Korean medicine diagnosis. This study aims to investigate the efficacy of YGJT on FD patients classified by 3-dimensional facial measurement using a 3-dimensional facial shape diagnostic system (3-FSDS).* Methods*. A placebo-controlled, double-blind, randomized, two-center trial will be performed to evaluate the efficacy of YGJT on FD patients. Eligible subjects will be initially classified as two types by 3-dimensional facial measurement using the 3-FSDS. Ninety-six subjects (48 subjects per each type) will be enrolled. These subjects will be randomly allocated into treatment or control groups in a 2 : 1 ratio. YGJT or placebo will be administered to each group during the 8-week treatment period. The primary outcome is total dyspepsia symptom scale, and the secondary outcomes include single dyspepsia symptom scale, proportion of responders with adequate symptom relief, visual analog scale, Nepean dyspepsia index-Korean version, functional dyspepsia-related quality of life, and spleen qi deficiency questionnaire.* Discussion*. This is the first randomized controlled trial to assess the efficacy of the YGJT on FD patients classified by 3-dimensional facial measurement. We will compare the treatment effect of the YGJT on FD patients classified as two types using the 3-FSDS. The results of this trial will help the FD patients improve the symptoms and quality of life effectively and provide objective evidence for prescribing the YGJT to FD patients in clinical practice.* Trial Registration*. This trial is registered with Clinical Research Information Service Identifier: KCT0001920, 15 May, 2016.

## 1. Introduction

Functional dyspepsia (FD) is a common functional gastrointestinal disorder characterized by chronic or recurrent abdominal discomfort or pain and symptoms, such as epigastric pain, epigastric burning, postprandial discomfort, and early satiety without evidence of organic diseases as confirmed by esophagogastroduodenoscopy (EGD) [1]. The prevalence of FD ranges from 11% to 29.2% worldwide [2]. In particular, more than 40% of patients who visit primary clinics and tertiary hospitals in South Korea have been diagnosed as having FD according to a survey using the Rome III criteria [3]. Various therapies for FD, including dietary modifications, antiemetics, antispasmodics, prokinetics, and analgesics, are commonly used as conventional treatments [4]. However, many patients with FD often seek out complementary and alternative therapies, such as herbal medicines and acupuncture, because of the limited effects of the conventional therapies [5–7].

Yukgunja-tang (YGJT; Rikkunshito in Kampo Medicine; Liu Jun Zi Tang in Traditional Chinese Medicine) is a herbal medicine comprising eight herbs; it is used to treat FD and relieve upper gastrointestinal symptoms, including dyspepsia, epigastric discomfort, and anorexia, in clinical practice in Korea, Japan, China, and other Asian areas [8–11]. In animal studies, YGJT improves relaxation of the gastric fundus, which maintains gastric storage capacity, and enhances gastric antral peristalsis, which facilitates stomach emptying. These effects improve gastric accommodation and emptying [12–16]. Some randomized controlled trials have been conducted to investigate the effect of YGJT on FD [17–21]. However, YGJT significantly improves only some symptoms of FD [18, 20]. In Korean medicine, FD is a common gastrointestinal disease that shows different treatment effects of herbal medicine depending on specific constitution or type of Korean medicine diagnosis. Accordingly, we will perform a randomized controlled trial to evaluate the efficacy of YGJT on FD patients classified as specific type by 3-dimensional facial measurement, which is one of the diagnostic methods of facial shape diagnosis in Hyungsang medicine.

Hyungsang medicine is a field of contemporary Korean medicine. It is based on the medical theories of Donguibogam, one of the most influential texts in Korean medicine. Hyungsang medicine emphasizes the fundamental constituents of humans and their constitutional differences and classifies persons into several types according to their outer appearances (shapes and colors) [22]. Facial shape diagnosis, a part of Hyungsang medicine, is conducted by observing facial characteristics like the shape and area of the face, along with the shape and location of the ear, eye, mouth, and nose. Facial shape diagnosis can be classified into several types including a bladder and a gallbladder body according to the aforementioned facial characteristics. For instance, people classified as bladder body have a wider frontal part of the face than the side part, and an overall round face and relatively big mouth. People classified as gallbladder body have a wider side part of the face than the frontal part, along with an angular face and relatively big nose [23, 24]. The theory of Hyungsang medicine states that if patients' outer appearances are different, the status of their inner organs is also different; therefore, even if the outer symptoms are the same, the treatments are different [22]. For example, YGJT, which will be used in this trial, is known to be more effective for the same symptoms in patients classified as the bladder than the gallbladder body [23, 25]. However, facial shape diagnosis, which is an important factor in Hyungsang medicine, has limitation; that is, it is performed through direct observation by clinicians. Thus, the diagnostic result tends to be subjective and inconsistent. The importance of a diagnostic device that can overcome the above limitation has recently been the focus to achieve more objective and standardized diagnosis [24, 26].

A 3-dimensional facial shape diagnostic system (3-FSDS) is a device for 3-dimensional facial measurement, and it is developed to overcome the limitations of the existing methods for the facial shape diagnosis. This device quantitatively measures the facial characteristics including the shape and area of the face, along with the shape and location of the ear, eye, mouth, and nose, and objectively classifies the type of facial shape as the bladder or the gallbladder body. The 3-FSDS is produced by Morpheus Co., Ltd. (Seongnam, Korea), with product manufacture approval from Korea Food & Drug Administration. Several studies have reported the development and clinical application of the 3-FSDS for 3-dimensional facial measurement [24, 27–31].

In this study, we will conduct a placebo-controlled, double-blind, randomized, two-center trial to demonstrate the efficacy of YGJT on FD patients classified as either bladder or gallbladder body by 3-dimensional facial measurement using the 3-FSDS and to verify the usefulness of the 3-FSDS for 3-dimensional facial measurement.

## 2. Materials and Methods

### 2.1. Study Design

This study will be conducted as a placebo-controlled, double-blind, randomized, two-center trial at the Kyung Hee University Korean Medicine Hospital in Seoul and the Dong-Eui University Korean Medicine Hospital in Busan, Korea, from July 2016 to November 2017.

**Figure 1:**
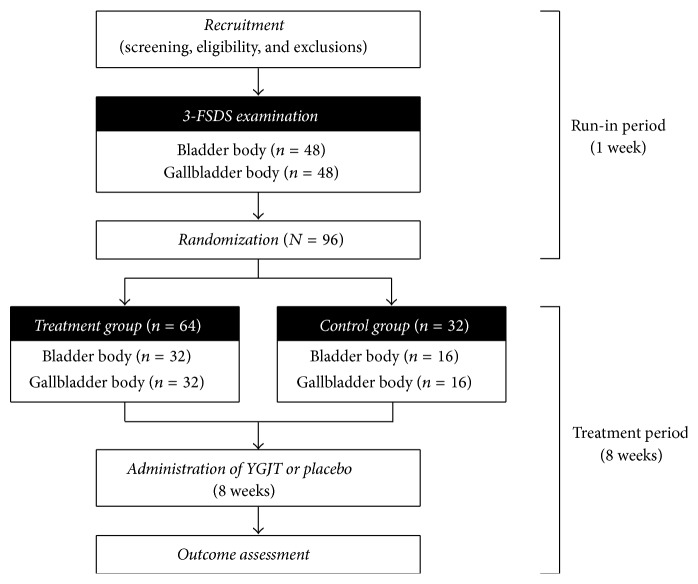
Flow chart of the trial. 3-FSDS, 3-dimensional facial shape diagnostic system; YGJT, Yukgunja-tang.

The trial will comprise a 1-week run-in period (week −1 to 0) and an 8-week treatment period (week 0 to 8). After screening and eligibility tests, subjects will be classified as specific type (“bladder body” or “gallbladder body”) using the 3-FSDS. A total of 96 subjects (48 bladder body, 48 gallbladder body) will be enrolled. The subjects will be randomized to treatment or control groups in a 2 : 1 ratio, including the same number of each subject type in each group, by an independent statistician. During the treatment period, 5.0 g of YGJT or placebo will be provided thrice a day (1 hour after each meal) for 8 weeks to the treatment or control groups, respectively. The trial flow is shown in Figure 1.

### 2.2. Participants

#### 2.2.1. Inclusion Criteria

Subjects who meet the following will be included: (1) subjects aged 19–75 years; (2) subjects who meet the Rome III criteria for FD; (3) subjects with more than 40 points on the visual analog scale (VAS; 0, no discomfort; 100, most severe discomfort) for the severity of dyspeptic symptoms; (4) subjects who agree to receive no other treatments during the study; (5) subjects who voluntarily agree with the study protocol and sign a written informed consent.

#### 2.2.2. Exclusion Criteria

Subjects who report any of the following will be excluded: (1) subjects with peptic ulcer or gastroesophageal reflux disease confirmed on EGD; (2) subjects with obvious signs of irritable bowel syndrome; (3) subjects with alarm symptoms, such as severe weight loss, melena, and dysphagia; (4) subjects with severe systemic organ diseases (cancer, diseases of heart, lung, liver, or kidney) or mental illness; (5) subjects who have had surgery related to the gastrointestinal tract, except for appendectomy more than six months ago; (6) subjects taking drugs that might affect the gastrointestinal tract; a minimum wash-out period of a week is required before participating in the study; (7) subjects who have had maxillofacial surgery or facial bone contouring surgery; (8) subjects who are pregnant or breastfeeding; (9) subjects who have malabsorption or maldigestion; (10) HIV positive subjects; (11) subjects with difficulties in taking part in the study (e.g., serious mental illness, dementia, drug addiction, time constraint, severe disorder in vision or hearing, and illiteracy); (12) subjects who have taken investigational drugs for other trials in the last three months.

### 2.3. Recruitment

Advertisements will be placed on the notice boards and homepages of the Kyung Hee University Korean Medicine Hospital and the Dong-Eui University Korean Medicine Hospital. The purpose, procedures, and potential risks and benefits of the study will be provided to the subjects in detail. All subjects should voluntarily sign written informed consent forms prior to enrollment.

### 2.4. Randomization, Allocation Concealment, and Blinding

Randomization will be separately performed at each center by an independent statistician. The subjects will be randomly assigned to the treatment or control group in a 2 : 1 ratio using block randomization method. This ratio is chosen because it will be more ethical to assign twice the number of subjects who will take placebo to the treatment group than to allocate equal number of subjects to each group [17]. Random numbers will be generated using the PROC PLAN of SAS 9.4 (SAS Institute Inc., Cary, NC, USA) by the independent statistician. The statistician will arrange the random numbers of each center in the two types (bladder body, gallbladder body) of facial shape and send the allocation lists to an independent staff. The staff will be in charge of the 3-FSDS program operation and know the type of facial shape of each subject.

When a subject passes the screening and eligibility test, his/her face image will be taken and stored using the 3-FSDS. Subsequently, the image will be sent to the independent staff operating the 3-FSDS program; the type of facial shape will be classified by 3-dimensional facial measurement using the 3-FSDS program. After the type is determined, the staff will immediately inform an investigator of the center through e-mail whether the subject will be included in the trial considering the fixed number of subjects for each type (24 bladder body, 24 gallbladder body at each center). If the subject is included in the trial, the staff will send a random number in the allocation list arranged in sequential order in each type to the investigator of the center through e-mail. Additionally, if the fixed number of subjects of each type is already full, the staff will notify the investigator that the new subject will be excluded from the trial.

The subjects, investigators, clinical research coordinator (CRC), and clinical pharmacist will be blinded to the random allocation of the trial. Only the independent statistician will be associated with the randomization. The independent staff operating the 3-FSDS program will know only the type of facial shape not the group allocation of each subject.

### 2.5. 3-Dimensional Facial Shape Diagnostic System (3-FSDS)

**Figure 2:**
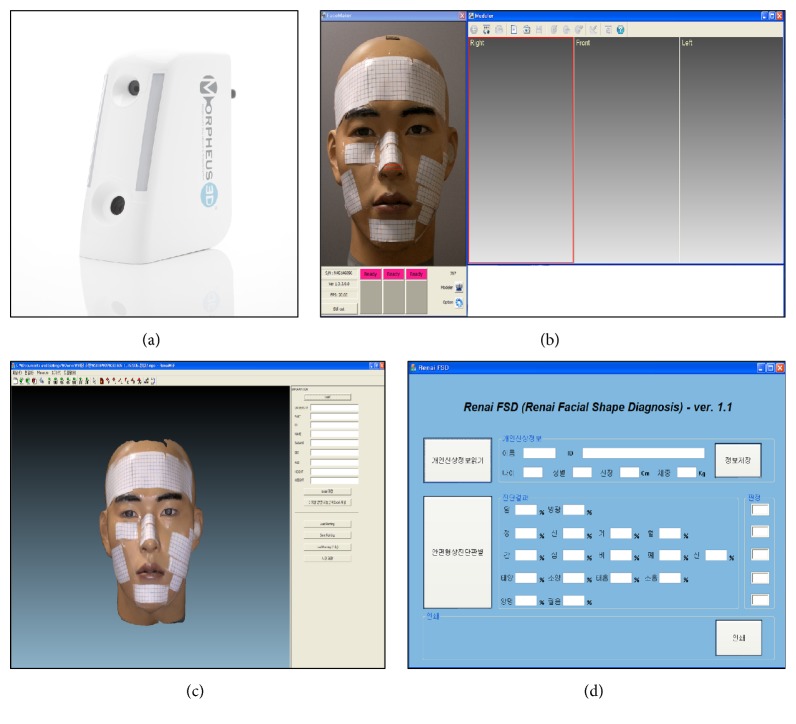
Components of 3-dimensional facial shape diagnostic system (3-FSDS). (a) Morphues 3D, (b) Real Face, (c) Renai MEF, and (d) Renai FSD.

The 3-FSDS comprises a 3D facial scanner (Morpheus 3D), scanner driving and data generation program (Real Face), 3D facial shape measurement program (Renai MEF), and 3D facial shape diagnosis program (Renai FSD) (Figure 2). The Morpheus 3D (Figure 2(a)) is a scanner for the 3D facial shape and is equipped with a camera (1024*∗*768, MV-CX37U, CREVIS), white LED light (5500 K), pattern generator, projector, and cooling fan. This scanner is convenient to operate and can capture 3D facial shape with a short scan time of only 0.8 s. It is safe to use because it does not harm the eyes [27]. The Real Face program (Figure 2(b)) controls the camera and the projector of the Morpheus 3D and captures the patterned projected images continuously at a high speed. The Real Face generates 3D data with acquired images through spatial coding, noise reduction filtering, 3D coordinate generations, mesh generations, and texture coordinate generations. The Renai MEF program (Figure 2(c)) automatically detects feature points and acquires the 3D coordinates of the points (including 39 points in the frontal part and 15 points in the side part of the face) from the 3D data. This program measures the length between the two points, angle of the three points, and area of the polygon on the eye, eyebrow, nose, and mouth parts with the images acquired by the Real Face and generates 337 variables. The Renai FSD program (Figure 2(d)) classifies the types of facial shape as the bladder or the gallbladder body after analyzing the variables obtained from the Renai MEF and calculating discriminant function. The analysis result is presented as percentage at each type, and the type with higher probability is considered as the final result of 3-dimensional facial measurement.

### 2.6. Interventions

After randomization, all subjects will be divided into the treatment (YGJT group) and control groups (placebo group). Each group will be provided with YGJT or placebo; the 5.0 g of YGJT or placebo will be taken thrice a day during the 8-week treatment period.

Brown bitter herbal extract YGJT granules (Yukgunja-tang granule®, Hankookshinyak Co., Ltd., Nonsan, Korea) produced in accordance with the Korean Good Manufacturing Practice guidelines will be used in this trial. Yukgunja-tang granule, a water-extracted YGJT mixed with cornstarch and lactose, is permitted and regulated by the Korean Food & Drug Administration. Each 5.0 g of YGJT granule contains* Pinelliae tuber* (1.33 g),* Citri unshii pericarpium* (1.33 g), Ginseng Radix Alba (1.33 g), Atractylodis Rhizoma Alba (1.33 g), Hoelen (1.33 g),* Glycyrrhizae radix* (0.50 g), Zingiberis Rhizoma Crudus (0.67 g), and* Zizyphi fructus* (0.67 g) as raw materials. All herbs will be obtained from qualified suppliers in Korea, and the YGJT granules will be sealed in opaque aluminum bags.

The placebo YGJT will be made with cornstarch and lactose with the same color and taste as the real YGJT by Hankookshinyak Co., Ltd., using the standard method of placebo manufacturing according to the Korean Good Manufacturing Practice guidelines. The placebo YGJT will be packed identically to the real YGJT in opaque aluminum bags with the same labeling.

The independent clinical pharmacist will distribute the investigational drugs to the subjects, and the CRC will ensure correct distribution. The subjects will be instructed to dissolve the YGJT or placebo granules in water and take them 1 hour after meals. The subjects are required to return the unused investigational drugs to the CRC at the next visit. The CRC and the clinical pharmacist will check the number of the returned investigational drugs and record it on a case report form (CRF). Treatment compliance will be evaluated at the end of the study by counting the number of the returned unused YGJT or placebo. The subjects with less than 70% compliance will be excluded from* per-protocol* analysis.

The subjects will not be allowed to take any concomitant medications associated with the treatment of FD during the trial. The subjects will also be instructed to report all prescribed or over-the-counter medications taken during the study at each visit and each telephone visit.

### 2.7. Outcome Measurements

#### 2.7.1. Primary Outcome

*Total Dyspepsia Symptom Scale (TDS Scale)*. The TDS scale comprises 8 items (postprandial fullness and bloating, early satiety, epigastric pain, epigastric burning, nausea, vomiting, belching, and other symptoms), with a 4-point Likert scale [17, 32]. The TDS score is the total score of 8 items. This scale will be assessed at baseline, 4 weeks, and 8 weeks.

#### 2.7.2. Secondary Outcomes

*(1) Single Dyspepsia Symptom Scale (SDS Scale)*. The SDS scale comprises three aspects of four principal symptoms of FD with a 4-point Likert scale [17, 32]. The symptoms are epigastric pain, epigastric burning, postprandial fullness and bloating, and early satiety. The three aspects are the frequency, intensity, and level of discomfort. The SDS score is the total score of the three aspects of the four symptoms. This scale will be evaluated at baseline, 4 weeks, and 8 weeks.

*(2) Adequate Relief (AR) of FD Pain and Discomfort*. The AR will be measured to assess the weekly improvement of the overall FD symptoms at each visit and each telephone visit during the treatment period. Subjects will be asked to answer the following question: “In the last week, have you had adequate relief of your pain or discomfort related to FD?” A proportion of responders (PR) will be assessed to compare the efficacy of the treatment. The responders will be defined as subjects reporting adequate relief for at least 50% of the treatment period, that is, responding “Yes” more than four times out of eight.

*(3) Visual Analogue Scale (VAS)*. The VAS measures the severity of overall dyspeptic symptoms (ranging from 0 mm as no discomfort to 100 mm as the most severe discomfort). The VAS will be measured at baseline, 4 weeks, and 8 weeks.

*(4) Nepean Dyspepsia Index-Korean Version (NDI-K)*. The Nepean dyspepsia index (NDI) [33, 34] is a reliable and validated disease-specific index for FD, which measures symptoms and health-related quality of life. The Korean version of NDI (NDI-K) validated by Lee et al. will be used [35, 36]. In this study, symptom-based questions, including the period, severity, and degree of distress of 15 symptoms, will be assessed using a 5- or 6-point Likert scale at baseline, 4 weeks, and 8 weeks. 

*(5) Functional Dyspepsia-Related Quality of Life (FD-QoL) Questionnaire*. The FD-QoL questionnaire measures the quality of life of FD patients. This questionnaire comprises four subscales of diet (5 items), daily activity (4 items), emotion (6 items), and social functioning (6 items) with a 5-point Likert scale. Higher total sum scores indicate worse quality of life. The FD-QoL questionnaire will be evaluated at baseline, 4 weeks, and 8 weeks.

*(6) Spleen Qi Deficiency Questionnaire (SQDQ)*. The SQDQ will be used to evaluate the spleen qi deficiency syndrome, which is the most common syndrome in FD patients [37, 38]. The questionnaire developed by Oh et al. [39] is composed of 11 items, and the total sum scores will be calculated weighing for each symptom. A cut-off point of the SQDQ to determine whether the subject is in a condition of spleen qi deficiency is 43.18 [40]. The SQDQ will be assessed at baseline, 4 weeks, and 8 weeks.

### 2.8. Safety and Adverse Event

The following tests will be performed to assess the safety at screening and 8 weeks: white blood cell, red blood cell, hemoglobin, hematocrit, platelet, aspartate aminotransferase, alanine aminotransferase, gamma-glutamyl transpeptidase, blood urea nitrogen, creatinine, and erythrocyte sedimentation rate. These tests will enable the investigators to exclude subjects with serious diseases before randomization and to verify the safety of the 8-week administration of YGJT in FD patients.

Adverse events (AEs) including serious AEs will be reported and recorded in detail during the entire study. An AE is any untoward medical occurrence that will not necessarily have a causal relationship with an investigational drug. Therefore, an AE can be any unfavorable and unintended sign (including an abnormal laboratory finding), symptom, or disease temporally associated with the use of the investigational drug, whether or not related to those. If any AEs occur, the appropriate treatment will be provided to the subject immediately. A serious AE will be defined as an event resulting in death, a life-threatening event, an illness requiring hospitalization, or persistent or significant disability. All of the serious AEs will be promptly reported to the institutional review board and the principal investigator within 24 hours.

### 2.9. Sample Size Calculation

The formula for estimating the sample size is as follows:

(1) N=nt+ncnt,the number of treatment group;nc,the number of control groupnc=12nt=Zα/2+Zβ2σ2λ+1/λμc−μt2.

A previous study demonstrated 1.57 points of improvement (*μ*_*c*_ − *μ*_*t*_ = *δ*) in the TDS scale for FD after 4 weeks of YGJT treatment compared with that after the placebo treatment [17]. The study indicated a mean standard deviation (SD = *σ*) of 2.148. In the present study, the ratio (*λ*) of the treatment group to the control group will be 2 : 1 (*λ* = 2). With a power (1 − *β*) of 80% and a significance level (*α*) of 5%, assuming *δ* = 1.57 and *σ* = 2.148, a sample size of *n*_*t*_ = 44 and *n*_*c*_ = 22 subjects will be required. Considering an assumed dropout rate of 30%, a total of 96 subjects will be needed in the current study.

### 2.10. Statistical Analysis

An independent statistician who is blinded to group allocation will conduct statistical analysis. All data will be presented as mean ± standard deviation or number (%). Baseline characteristics of the subjects will be analyzed using chi-squared test or Fisher's exact test for categorical variables and analysis of variance (ANOVA) for continuous variables. For the efficacy analysis, two-way ANOVA will be used to compare the changes in scores of each outcome for 8 weeks between the intervention groups or the facial shape types. For the safety analysis, the chi-squared test or Fisher's exact test will be used. Both* intention-to-treat* (ITT) and* per-protocol* (PP) analyses will be performed in the current trial, and the primary analysis will be based on the ITT analysis. For the ITT analysis, a full analysis set will be used, and all subjects randomly allocated to one of the two groups, have taken at least one dose of the investigational drugs, and have reported once at least the primary outcome after the baseline will be included. Missing data will be adjusted using the last observation carried forward method. For the PP analysis, all subjects who have completed the study and have complied well with the study protocol without major protocol violations will be included. The subjects with <70% compliance will be excluded from the PP analysis. For the safety analysis, all subjects who have taken at least one dose of the investigational drugs will be included. SPSS 21.0 (IBM SPSS Statistics, New York, USA) will be used for all statistical analyses. A* P* value < .05 will be considered statistically significant.

### 2.11. Quality Control

Regular monitoring will be conducted at each center by checking trial master files, informed consent forms, CRFs, compliance with treatments, AEs, and data records to ensure the accuracy and quality.

### 2.12. Ethics

The current study will be conducted in accordance with the standards of the International Committee on Harmonization of Good Clinical Practice and the revised version of the Declaration of Helsinki. The Institutional Review Board of the Kyung Hee University Korean Medicine Hospital (IRB number KOMCIRB-160115-HR-001) and the Dong-Eui University Korean Medicine Hospital (IRB number 2016-01) approved the trial protocol. This trial is registered at the Clinical Research Information Service (CRIS; KCT0001920; http://cris.nih.go.kr/cris/en/). Written informed consent will be obtained from all subjects prior to enrollment.

## 3. Discussion

This paper describes the protocol of a trial to investigate the efficacy of YGJT on FD patients classified by 3-dimensional facial measurement. This study is the first to explore the difference of the treatment effect of YGJT on FD patients classified as different types of facial shape using the 3-FSDS.

YGJT has widely been used to treat dyspeptic symptom of upper gastrointestinal diseases including FD in clinical practice in Korea, Japan, and China [8–10, 12]. A few animal and human studies have demonstrated that YGJT is effective on symptoms of FD. However, YGJT is reported to significantly improve only some symptoms of FD in clinical trials. Suzuki et al. [18] conducted a multicenter, double-blind, randomized, placebo-controlled, parallel-group trial to assess the efficacy and safety of YGJT on 247 FD patients. They showed that epigastric pain, an FD symptom, is significantly reduced by administration of YGJT. However, no significant differences were found on the proportion of patients with relief of other symptoms, such as epigastric burning, postprandial fullness, and early satiation, between the YGJT and placebo groups. In addition, a clinical trial with FD patients performed by Kusunoki et al. [20] reported that although the total score of the gastrointestinal symptom rating scale decreases after treatment with YGJT, the differences are not statistically significant. This finding may be caused by the treatment of FD, which is affected by specific constitution and type of Korean medicine diagnosis of patients. Differences of treatment effect of identical herbal medicine between FD patients with different constitutions or diagnosis types are possible. In traditional Korean medicine, particular facial characteristics have been believed to refer to assessable information related to an individual's diseases, and Hyungsang medicine has been developed as a constitutional medicine based on a Korean classical medicine literature, Donguibogam. According to the theory of Hyungsang medicine, the bladder body type has deficient qi and excessive dampness; on the other hand, the gallbladder body type has deficient Yin-blood and excessive heat. Therefore, YGJT is considered as a herbal medicine which is more effective to the bladder body type [22, 41]. Accordingly, we will examine the efficacy of YGJT on FD patients classified as bladder or gallbladder body by 3-dimensional facial measurement using the 3-FSDS.

In addition, we will evaluate the usefulness of the 3-FSDS comparing the treatment effect of YGJT, which is more effective on patients classified as the bladder than the gallbladder body in the theory of Hyungsang medicine, for FD patients classified as different facial shape types using the 3-FSDS. As mentioned above, the 3-FSDS is a diagnostic device developed to overcome the limitations of the existing methods for the facial shape diagnosis. If the symptoms of FD in this trial more significantly improve patients classified using the 3-FSDS as the bladder than the gallbladder body, then the 3-FSDS can be used to classify the different types of facial shape by 3-dimensional facial measurement.

The results of the current study are expected to demonstrate whether YGJT administration shows difference of treatment effect on FD patients depending on the facial shape types classified as bladder or gallbladder body using the 3-FSDS. If the efficacy of YGJT on FD patients classified as specific type is proven in this trial, their dyspeptic symptoms and quality of life will improve more effectively. The objective evidence for prescribing YGJT to FD patients diagnosed as specific facial shape type will also be provided with more confidence in clinical practice. In addition, if the usefulness of the 3-FSDS is verified, the 3-FSDS will be widely used in clinical practice as a device to diagnose facial shape type by 3-dimensional facial measurement more objectively. Furthermore, more objective diagnosis will aid in achieving a more effective treatment.
